# New Genes Involved in Osmotic Stress Tolerance in *Saccharomyces cerevisiae*

**DOI:** 10.3389/fmicb.2016.01545

**Published:** 2016-09-28

**Authors:** Ramon Gonzalez, Pilar Morales, Jordi Tronchoni, Gustavo Cordero-Bueso, Enrico Vaudano, Manuel Quirós, Maite Novo, Rafael Torres-Pérez, Eva Valero

**Affiliations:** ^1^Instituto de Ciencias de la Vid y del Vino – Consejo Superior de Investigaciones Científicas–Universidad de La Rioja–Gobierno de La RiojaLogroño, Spain; ^2^Departamento de Biomedicina, Biotecnología y Salud Pública, Universidad de CádizCádiz, Spain; ^3^Consiglio per la Ricerca in Agricoltura e l’Analisi dell’Economia Agraria-Centro di Ricerca per l’EnologiaAsti, Italy; ^4^Evolva Biotech A/SCopenhagen, Denmark; ^5^Departamento de Bioquímica y Biotecnología, Universitat Rovira i VirgiliTarragona, Spain; ^6^Departamento de Biología Molecular e Ingeniería Bioquímica, Universidad Pablo de OlavideSevilla, Spain

**Keywords:** osmotic stress, peroxisome, mitochondrial translation, Golgi-endosome, endomembrane system, GID-complex

## Abstract

Adaptation to changes in osmolarity is fundamental for the survival of living cells, and has implications in food and industrial biotechnology. It has been extensively studied in the yeast *Saccharomyces cerevisiae*, where the Hog1 stress activated protein kinase was discovered about 20 years ago. Hog1 is the core of the intracellular signaling pathway that governs the adaptive response to osmotic stress in this species. The main endpoint of this program is synthesis and intracellular retention of glycerol, as a compatible osmolyte. Despite many details of the signaling pathways and yeast responses to osmotic challenges have already been described, genome-wide approaches are contributing to refine our knowledge of yeast adaptation to hypertonic media. In this work, we used a quantitative fitness analysis approach in order to deepen our understanding of the interplay between yeast cells and the osmotic environment. Genetic requirements for proper growth under osmotic stress showed both common and specific features when hypertonic conditions were induced by either glucose or sorbitol. Tolerance to high-glucose content requires mitochondrial function, while defective protein targeting to peroxisome, GID-complex function (involved in negative regulation of gluconeogenesis), or chromatin dynamics, result in poor survival to sorbitol-induced osmotic stress. On the other side, the competitive disadvantage of yeast strains defective in the endomembrane system is relieved by hypertonic conditions. This finding points to the Golgi-endosome system as one of the main cell components negatively affected by hyperosmolarity. Most of the biological processes highlighted in this analysis had not been previously related to osmotic stress but are probably relevant in an ecological and evolutionary context.

## Introduction

Osmoregulation is fundamental for living cells, in order to prevent intolerable water flow rates through the plasma membrane, which would result in cell death. Osmoadaptation is primarily achieved by sensing and counterbalancing extracellular solute concentration. It becomes especially important for unicellular organisms, and even more so in the case of some microbial species that have evolved to grow on sugar rich environments, like the yeast *Saccharomyces cerevisiae*. Osmotolerance is particularly relevant for industrial wine yeasts. These yeast strains often thrive in the presence of 250–300 g/L sugar (glucose and fructose) as found in some batches of grape juice. Indeed, sugar concentration in grapes at harvest has been steadily increasing during the last 20 years mostly due to global climate warming. Industrial production of yeast biomass (for the bakery or wine industries) is in turn performed in molasses media, also inducing an important osmotic stress to yeast cells. Finally, increasing osmotic pressure by desiccation or by adding sugars or salts is still relevant for food preservation. Therefore, understanding microbial strategies and requirements to cope with osmotic stress is biotechnologically relevant.

*Saccharomyces cerevisiae* response to hyperosmotic stress involves arrest of cell-cycle progression, changes in gene expression at the transcription and translation levels, and singularly synthesis and retention of glycerol as a compatible osmolyte (Saito and Posas, 2012). The Hog1 MAP kinase plays a central role in orchestrating cell’s responses to high osmotic environment, through the high osmolarity glycerol (HOG) signaling pathway (Saito and Posas, 2012). This pathway has been extensively studied since it was discovered in 1993 (Brewster et al. 1993; Brewster and Gustin, 2014). Components of the pathway upstream and downstream Hog1 have been identified and characterized. It is able to regulate both acute responses and long term adaptation of yeast cells to osmotic stress (Brewster and Gustin, 2014). The identification of Hog1 orthologs in mammals, plants and other fungi has uncovered a remarkable functional conservation of stress activated MAP kinase pathways (Brewster and Gustin, 2014). Using the HOG pathway as a model system, has helped understanding not only osmoadaptation (Suescún-Bolívar and Thomé, 2015), but eukaryotic MAP kinases cascades, and signal transduction pathways at large (Saito and Posas, 2012).

Our current knowledge of HOG-based osmoregulation mechanisms in *S. cerevisiae* includes: (i) signaling upstream of Hog1, with two functionally redundant pathways (Sln1 and Sho1) converging to the MAP kinase kinase Pbs2, the specific activator of Hog1; (ii) additional elements required for nuclear translocation of phosphorylated Hog1; (iii) several negative feed-back mechanisms; (iv) interactions with other yeast MAP kinase cascades; (v) acute responses independent from transcriptional regulation (adjustment of metabolic flux, glycerol transport); (vi) targets and mechanisms of transcriptional regulation by Hog1; (vii) regulation of cell cycle progression (Saito and Posas, 2012).

Notwithstanding, our knowledge on the subtleties of yeast adaptation to hyperosmotic stress is continuously increasing, indicating that there are probably still many interesting features to be discovered. Some recent findings include for example, new partners of Hog1 for specific functions (Gonzalez-Novo et al., 2015), interactions of the HOG pathway with other regulatory pathways (Sharifian et al., 2015), or new mechanisms involved in transcriptional regulation by Hog1 (Nadal-Ribelles et al., 2014, 2015).

Whole genome analyses have certainly contributed to the elucidation of some of the features of adaptation to hyperosmotic stress in *S. cerevisiae*. In this context, yeast knock-out (YKO) collections constitute very interesting tools to analyze genetic requirements for yeast tolerance to specific environmental challenges. They provide information complementary to other whole genome techniques, like transcriptome analysis, since it has been shown that not all genes relevant for a biological process can be identified by their transcription profile, and conversely, a transcriptional response against a specific environmental condition of a given gene do not always indicate its relevance for adaptation to this condition (Birrell et al., 2002; Giaever et al., 2002; Tai et al., 2007). In the same line, Delneri et al. (2008) found that major controllers of growth rate are not under growth-rate dependent transcriptional control. Some authors have addressed yeast adaptation to hypertonic growth media (high-sucrose or high-glucose content) by using YKO collections, and high throughput strategies for the individual phenotyping of deletion strains (Ando et al., 2006; Teixeira et al., 2010). Also, sorbitol 1M was used as one of the stress conditions in quantitative fitness analysis experiments, based on microarray technology (Hillenmeyer et al., 2010). However, results of such experiments remained almost unexplored. In this work, we have identified some additional mechanisms of yeast adaptation to osmostress by quantitative fitness analysis of a diploid homozygous barcoded yeast deletion collection, using the Bar-seq technique.

## Materials and Methods

### Yeast Strain Collections

The homozygous and heterozygous yeast deletion strains, in the diploid BY4743 background, were purchased from Open Biosystems (Huntsville, AL, USA). Balanced pools for each collection were prepared on YPD broth (2% glucose, 2% peptone, 1% yeast extract) containing 200 μg/mL G418 (Sigma–Aldrich) and 15% glycerol, following the protocol described by Pierce et al. (2007). One mL aliquots of the yeast deletion strain pools were prepared in 2 mL screw-capped criotubes and stored at -80°C.

### Media and Culture Conditions

Control basal medium contained, per liter, Yeast Nitrogen Base w/o amino acids and ammonium sulfate, 1.7 g; (NH_4_)_2_SO_4_, 5 g; glucose, 30 g; histidine, 200 mg; leucine, 200 mg; uridine, 200 mg; pH 3.5. Osmotic stress by high-glucose was induced by increasing glucose concentration in the basal medium up to 200 g/L. The alternative osmotic stress condition was attained by including 0.6 M sorbitol in the basal medium.

Competition experiments of the genome-wide collections of mutants were performed under continuous culture conditions, in biological triplicates for each of the test media (control, high-glucose, and sorbitol), using small bioreactors MiniBio 250 mL (Applikon Biotechnology B.V., Delft, The Netherlands), with Rushton impellers. One mL of either the homozygote or heterozygote pool stored at -80°C was inoculated into 50 mL of YPD broth supplemented with 200 μg/mL G418 and incubated overnight at 28°C and 150 rpm. Bioreactors containing 150 mL of the medium were inoculated with this pre-culture to an initial OD_600_ of 0.2. Cultures were grown in batch mode during about 12 h prior to triggering the continuous cultures. In each case the same medium was used to launch the culture and in the feed reservoir. Continuous cultures were run for 10 generation times in the case of the homozygous collection, or 20 generation times for the heterozygous collection. Yeast cell samples were taken at the onset of the continuous cultures, and after the indicated numbers of generations, in order to compare pool compositions at the beginning and the end of the competition experiments. Dilution rate was stablished at 0.2 h^-1^. Temperature was set to 28°C, stirring to 500 rpm, and pH to 3.5 by automatic addition of NaOH 10 M.

### Bar-seq Analysis

Quantitative phenotyping via deep barcode sequencing (Bar-seq) was first described by Smith et al. (2009) as an improvement of the quantitative fitness analysis strategy previously developed by Giaever et al. (2002) and Pierce et al. (2007). In this work we have also incorporated continuous culture as in improvement in order to keep cells under almost constant conditions during the competition assays, as described previously (Delneri et al., 2008; Novo et al., 2013). In brief, the experiments consisted in growing pools for several generations in continuous culture, as described above, under control or osmotic stressed conditions. In order to estimate the abundance of each deletion strain in the mix before and after the competition experiments we took advantage of the 20 nt barcodes (up-tag and down-tag) associated to each deletion strain during the process of construction (Giaever et al., 2002), and flanking the geneticin resistance cassette. To this end, genomic DNA was extracted and PCR amplified using primers that bind to the conserved regions flanking each of the two barcode regions. The design of the primers facilitated further analysis via next-generation sequencing. Bioinformatics scripts were used to count the frequency of each tag in each biological replicate (three per growth condition). Further analysis was performed similar to standard transcription analysis based on RNA-seq, for example the edgeR package of Multi Experiment Viewer. However, we took into consideration counts are not affected by gene size, since we are calculating strain abundance, not actual gene expression. In this way it was possible to identify strains whose abundance was similar between control and stress conditions, as well as those that showed reduced or increased fitness under each of the stress conditions. Additional details are given below.

Several samples corresponding to 2 OD_600_ of cells were used for the genomic DNA extraction following the instructions described by manufacturer, DNeasy Plant Mini Kit (Qiagen). Purified genomic DNA was quantified by PicoGreen. A minimum of 7 ng of genomic DNA were employed as template for each PCR reaction, in order to avoid sample bias. The protocol was adapted to the current requirements of sequencing platforms as follows. Each 20-mer up- or down-tag barcode was amplified with combined primers comprised of the sequences of the common barcode primers and the sequences required for the sequencing using NextSeq from Illumina. For the up-tags the following primers were used: forward, 5′-**AATGATACGGCGACCACCGAGATCT***ACACTGACGACATGGTTCTACA****NNNNNN***GATGTCCACGAGGTCTCT-3′; reverse, 5′-**CAAGCAGAAGACGGCATACGAGAT** CTGCAGCGAGGAGCCGTAAT-3′. For the down-tags: forward, 5′- **AATGATACGGCGACCACCGAGATCT***ACACTGACGACATGGTTCTACA****NNNNNN***CGGTGTCGGTCTCGTAG-3′; reverse, 5′- **CAAGCAGAAGACGGCATACGAGAT**GGTATTGATAATCCTGATAT-3′. The 5′ portion (in bold) are the Illumina adaptors P5 and P7 sequences incorporated into the F and R primer, respectively. The custom adaptor sequence (italics) is required to sequence the Illumina multiplexing 6-mer indexing tags (bold italics). Finally, the underlined portion corresponds to the common primer flanking up- or down-tag sequences required to amplify the yeast barcodes.

After PCR amplification, products were purified, quantified and then adjusted to equal molar ratios to use as the Illumina sequencing template. Standard single-end sequencing primer was used, and sequencing was carried out with an Illumina NextSeq. The sequence reads have been deposited at the NCBI repository under the Sequence Read Archive SRP064889 and Bioproject PRJNA298959. The Illumina sequencing reads were assigned to different experiment samples using the 6-nucleotide multiplex index. Sequence reads were assigned to each tag by allowing two positon mismatches, by using a script in BioPython. This script counts the number of bases that are different between reads and each tag (mismatches), and counts the number of instances the rule “mismatches below 2” is satisfied for each tag. Total counts for each strain in each experiment/replicate were obtained by adding together counts for the corresponding up- and down-tags.

Different modules of Multi Experiment Viewer 4.9 were used for further analysis (Saeed et al., 2003, 2006). Clustering used the QTC method with the following parameters, maximum cluster diameter, 0.25; minimum cluster population, 25. Variations in strain abundance between test and control conditions, or among the two test conditions, were estimated by using the edgeR package (Robinson et al., 2010), with a specific annotation file designed to take into account the raw data do no refer to gene expression but to strain abundance (annotation file available upon request to the corresponding author). Inference algorithm for FDR calculation was common dispersion. Two actions were taken in order to reduce noise in the analysis of the competition experiments of the homozygous YKO collection. By one side strains represented by less than 10% of the average counts per strain, taking together samples from time zero and samples from the competitions in the basal medium (not under osmotic stress), were removed from the global analysis (430 strains were taken out this way). In addition, competitions with the heterozygous deletion collection were run for 20 generations in the basal medium, in conditions identical to those of the homozygous deletion collection. By comparing results of this competition under no osmotic stress between both collections, we identified numerous genes with an odd but common pattern of responses to competition conditions. Diploid strains carrying a single deletion of such genes were highly impaired, being quickly removed from the global population. In contrast, strains homozygous for the same gene knockout behaved close to the average, showing LogFC values around 0. It was considered that the phenotype of these heterozygous strains was more closely reflecting the actual function of the deleted gene. The phenotype of the corresponding homozygous strains was probably related to the selection of extragenic suppressors during propagation of the homozygous collection in the laboratory. This selection was probably driven by a strong phenotypic impact of the double deletion. Accordingly, any phenotype characterized in such homozygous strains would not truly reflect the function of the deleted gene. About 500 genes, with logFC > 0 in the homozygous collection without selective pressure, and logFC < -3 (FDR < 0.05) in the heterozygous collection without selective pressure, were considered as unreliable. In this way, a total of 927 genes were removed from the analyses described below. The overlap between the two gene lists mentioned above was very small (six genes).

The results of the pairwise comparisons between conditions are shown in **Data Sheet 1**. GO term enrichment was analyzed by using the YeastMine database (Balakrishnan et al., 2012). From each pairwise comparison, strains showing a Holm-Bonferroni adjusted *p*-value < 0.05, and a logFC < -1 or >1, were considered for GO enrichment analysis as underrepresented (DOWN) or overrepresented (UP) strains, respectively. GO terms were grouped in biomodules by GO/Module (Yang et al., 2011) to prioritize Gene Ontology terms. The only GO terms considered for discussion were those receiving a K (key) label in this analysis. Venn diagram was drawn by using Venny 2.1 on-line tool software (Oliveros et al. (2007–2015). String 10.0 (Franceschini et al., 2013) was used to visualize known interactions between the genes (or their gene products) deleted in the strains highlighted by the pairwise comparisons. Analysis and visualization parameters were as follows. Confidence level: 0.700; view mode, confidence; prediction methods, all except for text mining. The remaining statistical analyses were done using STATA-SE.

## Results

### Overview of Bar-seq Results

The phenotype of two YKO strains, deleted for either *HOG1* or *BUL1*, served as quality control references in this work. As mentioned above, the Hog1 MAP kinase plays a key role in the response of yeast cells to osmotic stress. Hog1 defective yeast strains are severely impaired under osmostress growth conditions. Accordingly, the homozygous strain deleted for YLR113W (*HOG1*) shows the most severe drop in relative abundance after ten generations with either glucose or sorbitol-induced osmotic stress (**Data Sheet 1**). Actually, tags associated to this strain completely disappeared from the yeast population in all the experimental replicates under osmotic stress (data not shown). On the other side, previous experimental evolution results revealed that Bul1 defective strains show a competitive advantage under continuous culture conditions in synthetic grape must media, in conditions similar to the experimental setup of the current work (Mangado et al., 2015). Results of the competition experiments performed in this work are in agreement with previous findings for Δ*bul1* strains. Homozygous deletion of YMR275C (*BUL1*) resulted in a significant increase in the relative proportion of the deleted strain in the population after 10 generations under osmotic stress, 1.3 to 1.6 LogFC (**Data Sheet 1**) respectively, for the comparison of sorbitol or high-glucose stressed populations against cells under no osmotic stress. The agreement of the present Bar-seq results with previous knowledge on the impact of *HOG1* or *BUL1* loss-of-function mutations on yeast tolerance to osmotic stress endorse the suitability of the experimental setup for identifying genes relevant for the adaptation of yeast cells to osmotic stress. Additionally, growth defects of Δ*slt2* or Δ*bck1* strains are known to be suppressed by increasing the medium osmolarity (Torres et al., 1991; Lee and Levin, 1992). Accordingly, the cognate deletion strains show improved fitness in the high-glucose competition; 1.3 and 1.1 LogFC, respectively (**Data Sheet 1**). However, in contrast to previous reports, the growth defect of these strains was not suppressed by sorbitol in this work, this is probably related to the osmolyte concentration that was 1 M in Torres et al. (1991) and Lee and Levin (1992), but only 0.6 M in our sorbitol assay.

**FIGURE 1:**
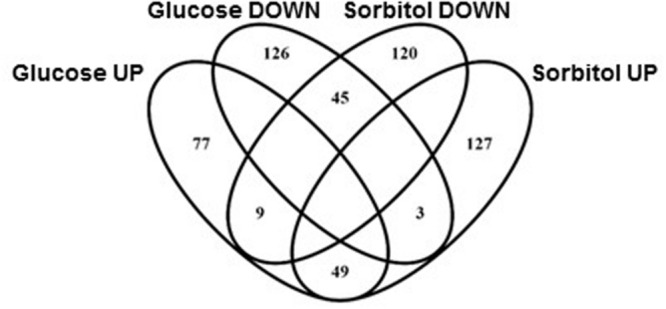
**Venn diagram showing overlapping between yeast knock-out (YKO) strains affected by either sorbitol or high-glucose osmotic stress (labeled DOWN) and strains whose fitness disadvantage was partially suppressed by sorbitol or high-glucose content in the medium (labeled UP)**.

LogFC values were calculated for the 4300 strains retained after applying the filters described in section “Materials and Methods.” This was done by pairwise comparison of relative strain abundances after ten generations in the basal medium or under each of the two stress conditions tested. Strains affected by the test growth conditions were identified using the thresholds described in section “materials and methods.” In addition to strains showing impaired growth under osmotic stress, this analysis unveiled a number of deletion yeast strains whose competitive fitness improved under hypertonic conditions (see below for further discussion on the meaning of these phenotypes). Results summarized in **Figure 1** clearly suggest there are common elements in the osmotic response to sorbitol or high-glucose content medium, both for genes required for optimal growth or gene deletions that benefit from these environmental conditions. This overlapping between results from the sorbitol or the high-glucose competition cultures involved about 25% of the strains highlighted in each analysis, and was statistically significant. There is also a short overlapping between strains favored in one condition and negatively affected in the other, but it was not statistically significant. The following sections will deal with the common and specific traits of gene requirements for optimal yeast growth under osmotic stress by high concentrations of sorbitol or glucose.

### Functional Enrichment among Genes Required for Optimal Growth under Osmotic Stress

**FIGURE 2:**
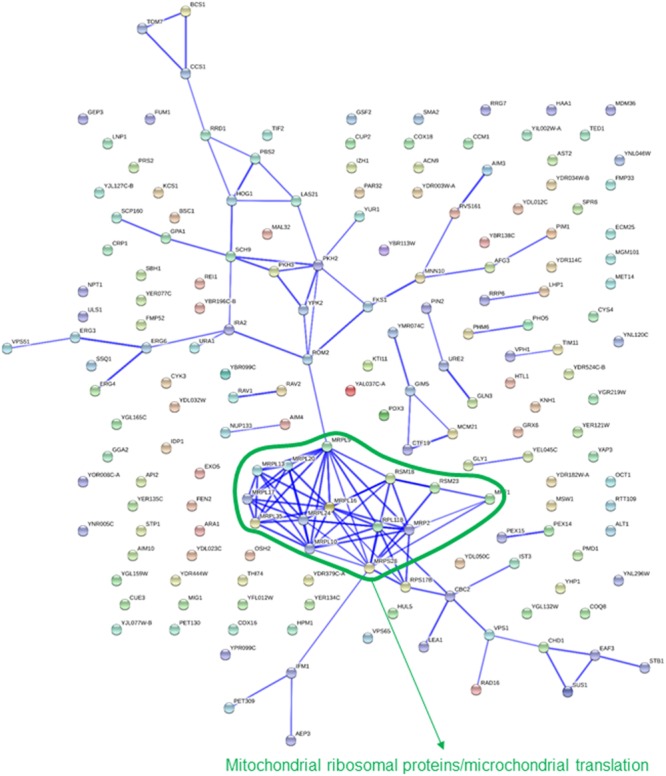
**Known interactions among genes or gene products highlighted by strains showing impaired growth under glucose-induced osmotic stress**. Image was obtained by using the String 10.0 application, with the parameters indicated in section “Materials and Methods.”

Most of the categories highlighted above were also pointed out by comparison of strains showing a significant change in abundance after 10 generations in either absence of selective pressure or under each of the osmotic stress conditions tested. A clear disadvantage for exponential growth under glucose-induced osmotic stress was identified for 174 strains. This set of genes was clearly enriched in categories related to mitochondria (**Table 1**). Most of these gene deletions showed little to no phenotypic impact in the assays using sorbitol, thus suggesting a specific requirement for mitochondrial activity. Additionally, the interaction network points also to mitochondrial functions (**Figure 2**), with a cluster of interconnected genes representing mitochondrial ribosomal proteins.

**Table 1:** GO enrichment for yeast knock-out (YKO) strains showing impaired growth under glucose-induced or sorbitol-induced osmotic stress.

Glucose	Biological process	Mitochondrion organization [GO:0007005]	9.5*E –* 5	30
		Cellular component organization [GO:0016043]	1.2*E –* 3	79
		
	Cellular component	Mitochondrial matrix [GO:0005759]	6.3*E –* 7	24
		Mitochondrion [GO:0005739]	1.4*E –* 3	55

Sorbitol	Biological process	Regulation of cellular metabolic process [GO:0031323]	4.6*E –* 5	60
		Negative regulation of gluconeogenesis [GO:0045721]	6.4*E –* 3	5
		Chromatin organization [GO:0006325]	2.6*E –* 2	24
		Single-organism cellular process [GO:0044763]	2.6*E –* 2	118
		
	Cellular component	GID complex [GO:0034657]	1.4*E –* 2	4

*Holm–Bonferroni adjusted *p*-values and number of positive hits are, respectively, shown in the two right-hand columns.*

**FIGURE 3:**
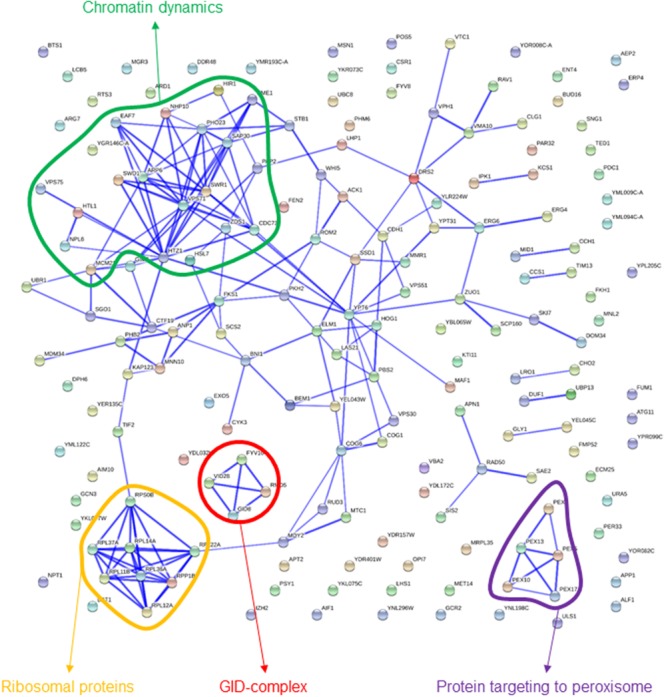
**Known interactions among genes or gene products highlighted by strains showing impaired growth under sorbitol-induced osmotic stress.** Image was obtained by using the String 10.0 application, with the parameters indicated in section “Materials and Methods.”

Some of the categories enriched among gene deletions affecting exponential growth in the presence of sorbitol are rather unspecific, providing an image that is not as clear-cut as for high-glucose above (**Table 1**). Notwithstanding, there are at least two terms that are obviously highlighted, “chromatin organization” and “GID complex,” the latter totally overlapping with “negative regulation of gluconeogenesis.” Both functions were also highlighted in the interactions network (**Figure 3**). In this image, a cluster of interconnected genes is joint to *HTZ1*, coding for a histone variant, H2AZ, involved in facilitating RNA Pol II passage. The four genes of this list coding for components of the GID complex are also clustered in **Figure 3**. This complex is involved in polyubiquitination and proteasome-dependent catabolite inactivation of the gluconeogenic enzyme fructose-1,6-bisphosphatase. In addition, there is a small cluster of ribosomal proteins that also seems to indicate one specificity of sorbitol as compared to glucose challenge. This was also revealed by direct comparison of results from the competition experiments between glucose and sorbitol-induced stress (see below). Finally, protein targeting to peroxisome, also highlighted by QTC analysis (see below), is represented by a well-defined group of genes in **Figure 3**.

### Functional Enrichment among Gene Deletions Rescued by Osmotic Pressure

The common trait of all the strains that show a competition advantage under some of the two types of osmotic stress tested (as compared to the unstressed condition) is they all decreased their relative abundance after 10 generation times in the basal medium. According to this observation, rather than “advantageous,” the associated gene deletions can still be considered as deleterious. However, their competitive disadvantage can be rescued by increasing concentrations of either glucose, sorbitol or both in the medium.

**FIGURE 4:**
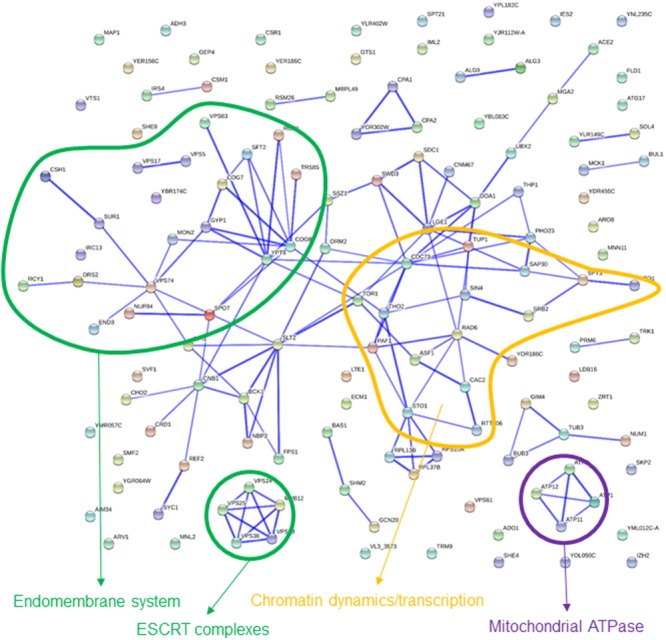
**Known interactions among genes or gene products highlighted by strains showing impaired growth in basal medium, but partially rescued in 20% glucose medium.** Image was obtained by using the String 10.0 application, with the parameters indicated in section “Materials and Methods.”

GO terms enriched among the 135 strains that were rescued by high glucose content involve several categories labeled as “negative regulation of metabolic process” (**Table 2**), as well as “regulation of lipid biosynthetic process” which, attending to the genes actually involved, is embedded on it. Most of these genes appear in the interaction map grouped with *TOR1, PAF1, TUP1*, and *ASF1* (**Figure 4**). Gene deletions affecting the “endomembrane system” are also clearly enriched among those 135 strains. Genes highlighting other related categories like those related to Golgi or endosome are all included among the 38 genes ascribed to this larger category. Most of these genes are interconnected in **Figure 4**. Although also falling under the “endomembrane system” term, five genes coding for components of the ESCRT-I, II, and III complexes form a specific cluster in the interaction network. This is in agreement with the corresponding GO terms in **Table 2** for Glucose. These complexes are involved in the sorting of monoubiquitinated proteins to the vacuole for degradation. The category “protein binding” includes in this case genes already included in the GO terms mentioned above, as well as *ATP11* and *ATP12*, which form a specific cluster with *ATP1* and *ATP2* in **Figure 4**. Those are structural proteins of the mitochondrial F1F0 ATP synthase (*ATP1* and *ATP2*) or required for its assembly (*ATP11* and *ATP12*). The pattern of abundance for these four gene deletions is striking. It decreases by 2–3 log (i.e., almost disappeared) after 10 generations in the basal or the sorbitol medium, clearly indicating a strong impairment. However, their competitive handicap was clearly (but partially) rescued by high glucose content in the medium. In addition, there were 179 strains rescued by sorbitol in this study. The portion of these genes that could be classified in enriched categories is small, but Golgi and endosome appear again as key words in this analysis (**Table 2**). This result suggests that the endomembrane system is specially affected by osmotic stress (see discussion below).

**Table 2:** GO enrichment for YKO strains showing impaired growth in basal medium, but partially rescued in 20% glucose, or 0.6 M sorbitol media.

Glucose	Biological process	Negative regulation of metabolic process [GO:0009892]	6.3*E –* 5	30
		Endosomal transport [GO:0016197]	1.4*E –* 3	13
		Regulation of lipid biosynthetic process [GO:0046890]	1.5*E –* 2	7
		
	Cellular component	ESCRT complex [GO:0036452]	3.1*E –* 3	5
		Endomembrane system [GO:0012505]	4.0*E –* 3	38
		Golgi apparatus part [GO:0044431]	9.2*E –* 3	15
		Golgi apparatus [GO:0005794]	1.7*E –* 2	17
		Endosome [GO:0005768]	3.2*E –* 2	13
		Endosome membrane [GO:0010008]	4.7*E –* 2	9
		
		Organelle subcompartment [GO:0031984]	4.7*E –* 2	9
		
	Molecular function	Protein binding [GO:0005515]	1.3*E –* 3	34

Sorbitol	Biological process	Protein localization to Golgi apparatus [GO:0034067]	4.1*E –* 3	6
		Protein retention in Golgi apparatus [GO:0045053]	1.7*E –* 2	5
		
	Cellular component	Endosomal part [GO:0044440]	3.9*E –* 5	15
		Endosome [GO:0005768]	2.4*E –* 4	18

*Holm–Bonferroni adjusted *p*-values and number of positive hits are, respectively, shown in the two right-hand columns.*

### Common and Specific Traits of the High-Glucose and Sorbitol Competitions

As indicated above only about one fourth of the strains impaired under one stress condition were also impaired in the other one (**Figure 1**). The same was true for strains rescued by high glucose or sorbitol content in the growth medium. No clear enrichment was found for specific GO terms among the 45 gene deletions impaired under both stress conditions. Nonetheless, *HOG1* appeared, as expected, as the most severely impaired deletion in both lists, while *PBS2*, a Hog1 MAPKK, also appeared in prominent positions. The only category enriched for the 49 gene deletions indifferently rescued by high-glucose or sorbitol is “Retromer complex” (data not shown), highlighted by *VPS5* and *VPS17*.

As expected, the list of genes behaving completely opposite under high-glucose or sorbitol conditions (i.e., impaired in one condition and rescued in the other as compared to the basal medium) was much shorter, three strains among those impaired under high-glucose-induced osmotic stress, including *GLN3*, and nine among those impaired by sorbitol stress, including two components (*SAP30* y *PHO23*) of the Rpd3L complex. Nevertheless, direct comparison of the original Bar-seq data between hypertonic glucose and sorbitol continuous cultures shed some more light on the differences between these growth conditions in terms of gene activity requirements for *S. cerevisiae*. Above 500 strains showed differential abundances between the two competitions (**Table 3**). The results are mostly confirmatory of the biological functions or structures already highlighted for each growth condition individually. According to **Table 3** some processes involved in chromatin remodeling, in relation with transcriptional control, including *HTZ1* or the Rpd3L complex, would be more specifically rescued by glucose; while, proper mitochondrial function would be more necessary to cope with glucose challenge than osmotic stress induced by sorbitol (**Table 3**).

**Table 3:** GO enrichment for YKO strains showing reduced (DOWN) or improved (UP) growth under sorbitol-induced as compared to glucose-induced osmotic stress.

UP	Biological process	Negative regulation of biosynthetic process [GO:0009890]	3.9*E –* 8	38
		Biological regulation [GO:0065007]	4.8*E –* 5	98
		Negative regulation of nucleic acid-templated transcription [GO:1903507]	6.8*E –* 4	27
		Negative regulation of RNA metabolic process [GO:0051253]	8.5*E –* 4	27
		Negative regulation of cellular carbohydrate metabolic process [GO:0010677]	4.4*E –* 2	6
		
	Cellular component	Protein complex [GO:0043234]	6.8*E –* 4	77
		Chromatin [GO:0000785]	6.9*E –* 3	16
		Rpd3L-Expanded complex [GO:0070210]	1.3*E –* 2	6
		Rpd3L complex [GO:0033698]	1.6*E –* 2	5

DOWN	Biological Process	Cellular component organization [GO:0016043]	2.6*E –* 4	117
		Mitochondrial respiratory chain complex assembly [GO:0033108]	2.3*E –* 3	10
		Mitochondrion organization [GO:0007005]	5.8*E –* 3	36
		
	Cellular component	Mitochondrial part [GO:0044429]	1.2*E –* 3	50

*Holm–Bonferroni adjusted *p*-values and number of positive hits are, respectively, shown in the two right-hand columns.*

### Cluster Analysis of Competition Experiments

**FIGURE 5:**
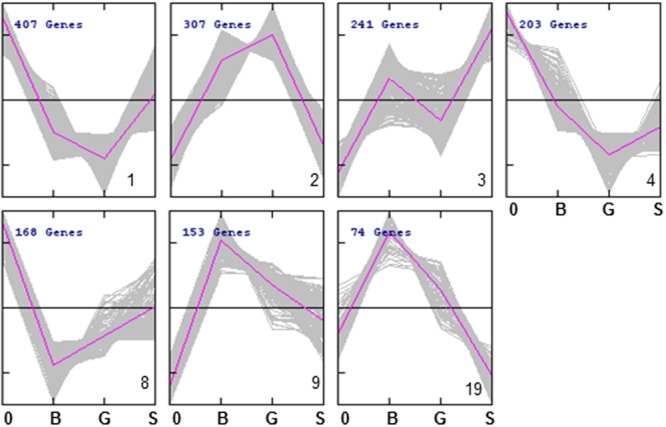
**Abundance profiles of YKO strains in the 7 QTC clusters (out of 26) showing significant enrichment for at least one GO term.** Refer to **Data Sheet 2** for a complete view of the 26 clusters.

We used cluster analysis as a complementary approach in order to draw biological information from the competition experiments. This analysis grouped the strains into 26 different clusters (**Figure 5**; **Data Sheet 2**). The different patterns of relative abundance distribution cover almost all the possibilities, including gene deletions resulting in impaired growth under all growth conditions (e.g., cluster 4), those affecting survival under both glucose or sorbitol-induced osmotic stress (e.g., cluster 10); or strains whose defective growth seems to be rescued by osmotic pressure, among other options. The gene lists associated to each cluster were explored for GO term enrichment. Only seven clusters showed a significant enrichment in this analysis.

Strains in clusters 1 and 4 show a general trend to reduced strain frequency under all growth conditions, as compared to frequencies before competition. In addition, high-glucose further impairs their competitive fitness. In both cases sorbitol seems to be less harmful than glucose, although they differ in whether they compete in sorbitol better than in the basal medium (cluster 1) or not (cluster 4). GO enrichment is also similar for both clusters (**Table 4**). About half of the strains under the GO term “mitochondrion” in **Table 1** are listed under the same label in either Cluster 1 or 4. This supports the previous conclusion of mitochondrial function being relevant for survival under osmotic stress, but mostly when this stress is generated by high-glucose concentration. Cluster 2 groups strains specifically affected by sorbitol (**Figure 5**), and is enriched for gene deletions affecting constituents of the external membrane (**Table 4**). In contrast strains in Cluster 3 show competitive advantage in sorbitol medium (**Figure 5**) and are enriched for strains defective in vacuolar function. This suggest components of the cell periphery must be specially required to survive sorbitol challenge, while vacuolar functions would be specific targets for this stress conditions. Cluster 8, enriched for dynein complex, does not seem very specific of osmotic stress. Protein targeting to peroxisome is enriched in cluster 9, with strains impaired under osmotic stress, but more strongly in the case of sorbitol. Importance of this function to survive sorbitol challenge was already highlighted in **Figure 3**. Cluster 19, grouping strains strongly affected by sorbitol, is enriched for HIR complex, related to “chromatin organization,” a category already seen in **Table 1**; **Figure 3**.

**Table 4:** GO enrichment for YKO strains grouped in the different clusters identified by the QTC method grouped by cluster number as shown in **Figure 5**.

1	Biological process	Mitochondrion organization [GO:0007005]	4.0*E –* 5	50
		Mitochondrial respiratory chain complex assembly [GO:0033108]	5.3*E –* 3	11
		Regulation of mitochondrial translation [GO:70129]	2.8*E –* 2	7
		
	Cellular component	Mitochondrial ribosome [GO: 0005761]	5.1*E –* 7	22
		Mitochondrion [GO:0005739]	1.5*E –* 3	99
		Mitochondrial inner membrane [GO: 0005743]	4.6*E –* 2	29

2	Cellular component	Cell periphery [GO: 0071944]	1.0*E –* 2	56
		Anchored component of membrane [GO: 0031225]	1.5*E –* 2	12
		Extracellular region [GO: 0005576]	2.5*E –* 2	16

3	Cellular component	Vacuole [GO: 0005773]	4.9*E –* 3	42
		Intrinsic component of membrane [GO: 0031224]	4.9*E –* 2	84

4	Biological process	Mitochondrion organization [GO:0007005]	4.1*E –* 12	47
		Mitochondrial DNA metabolic process [GO:0032042]	1.3*E –* 2	5
		
	Cellular component	Mitochondrial part [GO:0044429]	7.9*E –* 13	57
		Mitochondrion [GO:0005739]	4.2*E –* 5	78
		Ribosomal subunit [GO:0044391]	2.2*E –* 3	22
		
	Molecular function	Structural constituent of ribosome [GO:0005198]	4.9*E –* 3	28

8	Cellular component	Dynein complex [GO: 0030286]	2.2*E –* 2	4

9	Biological process	Transmembrane transport [GO:0055085]	4.4*E –* 4	27
		Protein targeting to peroxisome [GO:0006625]	4.3*E –* 2	6
		
	Cellular component	Peroxisomal importomer complex [GO:1990429]	3.8*E –* 2	4
		
	Molecular function	Transporter activity [GO:0005198]	4.8*E –* 2	23

19	Cellular component	HIR complex [GO:0000417]	4.5*E –* 3	3

*Only clusters showing at least one significantly enriched category are shown. Holm–Bonferroni adjusted *p*-values and number of positive hits are, respectively, shown in the two right-hand columns.*

## Discussion

Osmotic stress response in *S. cerevisiae* has been a workhorse to improve our understanding of cell adaptation to changing environmental conditions, and MAP kinase signal transduction pathways, that are greatly conserved among eukaryotic organisms. The yeast Hog1 MAP kinase and its homologs in other eukaryotic species are part of complex signaling networks that coordinate multiple processes required to ensure cell survival under often aggressive environments. Cell adaptation to osmotic stress is a multidimensional process, so that, after years of intensive research, from the discovery of *HOG1* more than 20 years ago (Brewster et al., 1993), new findings are still continuously arising (Nadal-Ribelles et al., 2014; Dexter et al., 2015; Gonzalez-Novo et al., 2015; Sharifian et al., 2015). In this context, yeast genome-wide phenomic screens can contribute to better explain the subtleties of cell responses to osmotic stress. Genome-wide analysis of the genetic requirements for yeast tolerance to osmotic stress have been mostly based on the analysis of the phenotypes of individual strains (Ando et al., 2006; Teixeira et al., 2010).

Some of the results of the fitness analysis performed in this work are confirmatory of previous knowledge. However, there is very little overlapping between genes identified in the current study as required for a good fitness under osmotic stress and those found in other recent genome wide studies (Ando et al., 2006; Teixeira et al., 2010). One important difference in the experimental setup is previous works assessed the impact of osmotic stress by phenotyping isolated strains in batch mode, while the current work was based on competition experiments in continuous culture. Indeed, above 75% of the gene deletions identified as relevant for osmotolerance by Ando et al. (2006) or Teixeira et al. (2010) had been filtered out from the current study because they were almost depleted in the competition assays even under non-hypertonic conditions (data not shown). In contrast, gene deletions identified in competition experiments as non-dispensable under hypertonic conditions will probably show milder phenotypes in direct assays (i.e., phenotyping of isolates strains), and not be taken into consideration. In conclusion, the direct and the competition approaches are complementary, since the set of informative genes is essentially different.

One particularity of quantitative fitness analysis is the identification of yeast deletion strains apparently showing a better fitness under osmotic pressure than under standard conditions. However, a close-up view of the data does not support the simple conclusion that these gene deletions are advantageous for yeast cells, rather the opposite. In most cases, these cell functions might be strongly damaged in normal cells under hypertonic conditions. In relative terms, deletion strains already impaired in these pathways would be less affected by the stress conditions, resulting in an apparent gain of fitness as compared to basal medium.

Despite a significant overlapping between glucose and sorbitol-induced stress conditions, we found specific features for each one of these osmolytes. This is not surprising, since specificity of the cell responses to particular osmotic stress conditions has been described in several studies. For example, different concentrations of NaCl result in different temporal patterns in nuclear accumulation of Hog1 (Hohmann, 2015). Gomar-Alba et al. (2015) found a slower Hog1 delocalization, as well as higher *HOT1* expression, under high-glucose stress than under other osmostress conditions, including sorbitol and NaCl. Together with previous results by Capaldi et al. (2008) these authors suggest inhibition of Msn2/4 by high glucose concentrations results in an overall decrease in the activation of the general stress response, and a shift of the Hog1 dependent expression program toward specific subsets of genes. Interconnection between HOG and other signaling pathways, including the pheromone or the Msn2/4, have recently been demonstrated by phosphoproteome analysis (Vaga et al., 2014; Kanshin et al., 2015). Also Dihazi et al. (2004) found different patterns of Pfk2 phosphorylation in response to either NaCl or hyperosmolar glucose. This was related to the glucose dependent activation of the Ras-cAMP pathway, in addition to the HOG pathway. A specific relation between glucose availability and activation of the HOG MAPK signaling cascade has recently been described by Sharifian et al. (2015), although the relevance of this link for the present work is uncertain, given that glucose was always present in sufficient amounts (as carbon and energy source), independent of the osmolyte used to induce hypertonic conditions.

Proper mitochondrial function is revealed here as a critical factor for yeast adaptation to osmotic stress, specifically when glucose is the stress-inducing agent. Interestingly, Martinez-Pastor et al. (2009) also found mitochondrial functions to be required for salt stress tolerance. They identified up-regulation of mitochondria as an essential feature of yeast adaptation to hyperosmotic stress, through the reduction of reactive oxygen species. According to our results, this seems to be true also for glucose-induced stress, but is probably less relevant in the case of sorbitol. Our result might also be related to those by, Ando et al. (2006) who identified ATP depletion as a relevant factor in sensitivity to high-sucrose stress.

On the other side, “protein targeting to peroxisome” functions appear as non-dispensable to tolerate sorbitol-induced osmotic stress. The involvement of peroxisome in osmotic stress tolerance might be related to the contribution of this subcellular compartment to the detoxification of reactive oxygen species (Lopez-Huertas et al., 2000). Also noteworthy is the fact that peroxisomal localization of several proteins, including Gpd1, depending on environmental conditions, might play a role in cell survival through the spatial regulation of redox potential (Jung et al., 2010).

Other functions that seem to be specifically required for survival under sorbitol but not under glucose-induced stress are “chromatin organization” and “GID complex” or “negative regulation of gluconeogenesis” (**Table 1**). It is noteworthy that chromatin remodeling has been specifically related to osmostress induced gene expression; which is prevented by monomethylation of H3K4 (Nadal-Ribelles et al., 2015). On the other side, the relevance of an effective negative control of gluconeogenic activities to tolerate osmotic stress might be related to the carbon flux, since the activity of fructose-1,6-bisphosphatase will compete with the glycerol biosynthesis pathway for the intermediary metabolite fructose-1,6-bisphosphate. Indeed, one of the targets of the HOG pathway is 6-phosphofructo-2-kinase (Dihazi et al., 2004), whose activation results in the stimulation of the upper part of glycolysis, in turn required to feed DHAP for glycerol biosynthesis (Brewster and Gustin, 2014).

In addition to identifying gene functions relevant for optimal growth under osmotic stress, the current quantitative fitness analysis pinpointed to gene deletions whose impaired fitness was at least partially rescued by increasing the osmolarity of the medium. The picture arising by taking together results from cultures challenged with glucose or sorbitol points to the endomembrane system as one of the main cellular functions rescued under hypertonic conditions. For some hits, this might be related to actual osmotic remedial, like as described for mutants in the PKC1 pathway (Torres et al., 1991; Lee and Levin, 1992), or in endocytosis (Aghamohammadzadeh and Ayscough, 2009). However, this mechanism does not seem to account for the apparent rescue of most of the mutant strains in this analysis. Our conclusion is that the Golgi-endosome system is one of the cell components more severely impaired under osmotic stress. Competition under stress will bring closer the survival chances of normal cells and those genetically defective. A recent screening of differential genetic interactions of yeast MAPK pathway genes also suggest a role for membrane related protein complexes in tolerance to sorbitol stress (Martin et al., 2015), including the retromer complex, as well as SNARE proteins (involved in membrane vesicle docking and membrane fusion processes). In addition, phosphoproteome dynamics studies points to several steps of endosome maturation as targets of osmotic shock (Kanshin et al., 2015).

In summary, through an advanced quantitative fitness analysis approach, we have identified novel biological processes that are involved in the interaction of yeast cells with the osmotic environment. Fitness analysis involved the use of yeast knock-out collections, continuous culture, and Bar-seq. In contrast to most findings from previous genome-wide studies, most of the genes identified in the present work as non-dispensable for survival under osmotic stress do not show a marked fitness impairment in the basal medium, making this work complementary to previous ones. In agreement to previous studies, we found the sets of genes required for a good adaptation to osmotic stress are only partly overlapping depending on the nature of the osmolyte considered. Appropriate mitochondrial function appears as a clear requirement for yeast survival under glucose-induced stress, while optimal growth kinetics under sorbitol-induced osmotic stress seems to rely on proper peroxisomal function and inhibition of gluconeogenesis (probably through competition for glycolytic intermediates). Some elements involved in transcriptional control and chromatin remodeling also appear as non-dispensable under sorbitol challenge. The particularities of the present analysis also allowed to identify the Golgi-endosome system as potential specific targets of osmotic induced damage in the cell. These new findings show the potential of the proposed combination of techniques to enlarge our knowledge on seemingly well-known biological processes, as osmotic stress tolerance. Some of the new targets and survival mechanisms to osmotic challenge might be difficult to recognize by direct assays, but seem to be relevant in a competition context, which makes more sense in ecological and evolutionary terms.

## Author Contributions

RG, PM, and EvV conceived and designed the experiments. GC, EnV, MQ, and MN performed the experiments. RG, JT, RT, analyzed the data. RG, JT, PM, and EvV interpreted the results. All authors discussed and approved the manuscript.

## Conflict of Interest Statement

The authors declare that the research was conducted in the absence of any commercial or financial relationships that could be construed as a potential conflict of interest.

## Supplementary Material

DATA SHEET S1 **Excel file summarizing the logFC values obtained from the pairwise comparison of competition experiments for the 4300 YKO strains tested**.

DATA SHEET S2 **Abundance profiles of YKO strains grouped in each of the 26 clusters obtained by the QTC method.** Growth conditions are time *t* = 0 (0), no osmotic stress (B), glucose (G), and sorbitol (S)-induced osmotic stress.
