# Supplementary figures and images for: New Genes Involved in Osmotic Stress Tolerance in Saccharomyces cerevisiae
Source: Front Microbiol. 2016 Sep 28;7:1545. doi: 10.3389/fmicb.2016.01545 (PMC5039201; doi:10.3389/fmicb.2016.01545)

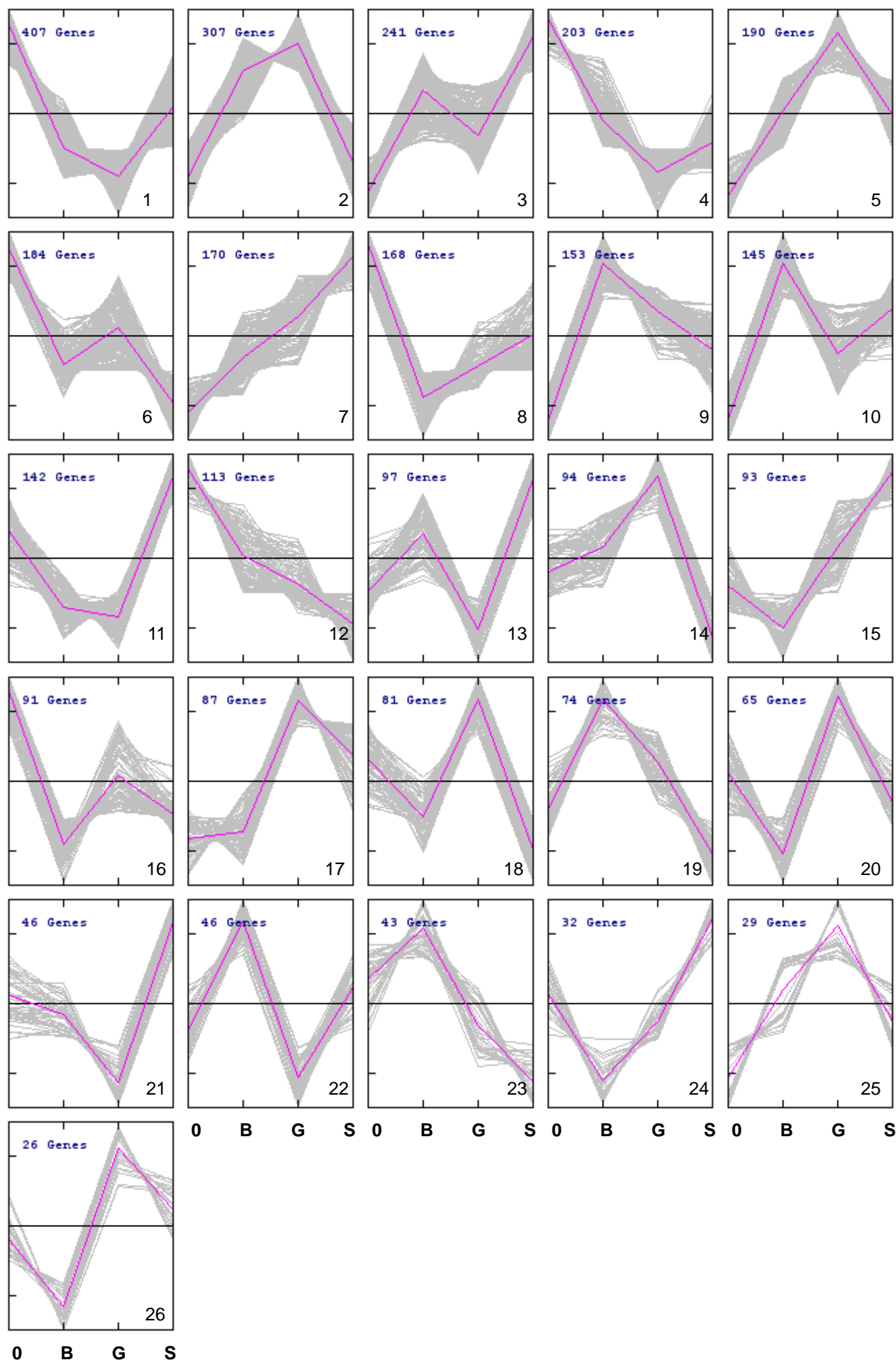

Supplement: DATA SHEET S2 — Abundance profiles of YKO strains grouped in each of the 26 clusters obtained by the QTC method. Growth conditions are time t = 0 (0), no osmotic stress (B), glucose (G), and sorbitol (S)-induced osmotic stress. [file Data_Sheet_2.PDF]
